# Adaptation of controlled attenuation parameter (CAP) measurement depth in morbidly obese patients addressed for bariatric surgery

**DOI:** 10.1371/journal.pone.0217093

**Published:** 2019-05-24

**Authors:** Sosthene Somda, Amandine Lebrun, Hadrien Tranchart, Karima Lamouri, Sophie Prevot, Micheline Njike-Nakseu, Martin Gaillard, Panagiotis Lainas, Axel Balian, Ibrahim Dagher, Gabriel Perlemuter, Sylvie Naveau, Cosmin Sebastian Voican

**Affiliations:** 1 Faculté de Médecine Paris-Sud, Univ Paris-Sud, Université Paris-Saclay, Le Kremlin-Bicêtre, France; 2 Service d’Hépato-Gastroentérologie et Nutrition, Hôpital Antoine-Béclère, Hôpitaux Universitaires Paris-Sud, Assistance Publique-Hôpitaux de Paris, Clamart, France; 3 INSERM U996, DHU Hepatinov, Labex LERMIT, Clamart, France; 4 Service de Chirurgie Digestive Minimale Invasive, Hôpital Antoine-Béclère, Hôpitaux Universitaires Paris-Sud, Assistance Publique-Hôpitaux de Paris, Clamart, France; 5 Service d’Anatomie pathologique, Hôpital Antoine-Béclère, Hôpitaux Universitaires Paris-Sud, Assistance Publique-Hôpitaux de Paris, Clamart, France; Medizinische Fakultat der RWTH Aachen, GERMANY

## Abstract

**Background and aim:**

The controlled attenuation parameter (CAP) using FibroScan (Echosens, Paris, France) M or XL probe has been developed for liver steatosis assessment. However, CAP performs poorly in patients with high body mass index. The aim of our study was to assess whether CAP is overestimated using the standard XL probe in patients with morbid obesity, and in the case of an overestimation, to reprocess the data at a greater depth to obtain the appropriate CAP (CAPa).

**Patients and methods:**

We conducted an observational prospective cohort study on a total of 249 severely obese patients admitted to our institution to undergo sleeve gastrectomy. Patients had a liver biopsy performed during the surgery and a CAP measurement during the 15 days preceding biopsy. Patient files were reprocessed retrospectively by an algorithm, blinded to the patients’ clinical data. The algorithm automatically assessed the probe-to-capsula distance (PCD) by analysing the echogenicity of ultrasound signals on the time-motion mode. In the case of a distance >35 mm, the algorithm automatically selected a deeper measurement for CAP (CAPa). When PCD was less than 35 mm, the measured CAP was considered as appropriated (CAPa) and no further reprocessing was performed.

**Results:**

CAP recording was not performed at a sufficient depth in 130 patients. In these patients, the CAPa obtained at the adapted depth was significantly lower than CAP (298±3.9 versus 340±4.2 dB/m; p< 0.0001) measured at the standard depth (35 to 75 mm). Multiple linear regression analysis revealed that both body mass index and hepatic steatosis were independently correlated with CAP values. After reprocessing the CAP in patients with PCD > 35 mm, steatosis stage was the only parameter independently correlated with CAP values. For the diagnosis of steatosis (S≥1), moderate to severe steatosis (S≥2) and severe steatosis (S = 3), the AUROC curves of CAPa (measured CAP in patients with PCD<35 mm and reprocessed CAP in those with PCD>35 mm) were 0.86, 0.83 and 0.79, respectively. The Obuchowski measure for the diagnosis of steatosis was 0.90±0.013.

**Conclusion:**

CAP was overestimated in a half of morbidly obese patients using an XL probe, but CAP can be performed correctly in these patients after adapting the measurement depth.

## Introduction

Non-alcoholic fatty liver disease (NAFLD) is already a major public health issue with an estimated worldwide prevalence between 25% in general population and more than 80% in populations with obesity and metabolic syndrome [1]. Its burden is expected to rise in the context of ongoing increase of obesity prevalence. NAFLD comprises histological lesions ranging from pure steatosis (NAFL) to steatosis plus necroinflammation (NASH, non-alcoholic steatohepatitis) with or without fibrosis [2]. Steatosis is generally associated with a benign outcome and no mortality increase compared to general population. In contrast, evolution to NASH stage increases the risk of progression to advanced liver disease (cirrhosis and/or hepatocellular carcinoma) and mortality [3]. The presence of steatosis is a mandatory finding to support the diagnosis of NAFLD. An accurate diagnosis of steatosis is therefore important for early identification of NAFLD in risk populations. Liver biopsy is considered to be the gold standard for the evaluation of steatosis. Nevertheless, liver biopsy is an invasive test with risk of complications and low acceptability, unsuitable for screening. Furthermore, steatosis severity may change within just a few weeks of therapeutic intervention, and liver biopsy cannot be suitable for patient follow-up. Therefore, a number of imaging technics have been developed and provide potential alternatives for the diagnosis of steatosis.

Magnetic resonance (MR) based techniques assess triglyceride specific signal intensity and are sensitive approaches for non-invasive steatosis detection, but their use is limited by the high costs and the poor comparability between different MR technics [4]. Conventional B-mode ultrasonography (US) is the most commonly used imaging technic to detect steatosis, with a very good specificity (>90%) and sensibility (>80%) when used by a trained operator [5]. However, US can only detect moderate to severe steatosis (≥20–30%) and is operator-dependent. All these limitations could potentially be overcome by the use of controlled attenuation parameter (CAP) feature. CAP is determined by measuring the degree to which the ultrasound signal is attenuated by hepatic fat at the central frequency of the transient elastography [FibroScan (Echosens, Paris, France)] M or XL probe while a liver stiffness measurement (LSM) is being obtained. CAP has been reported to perform well for mild steatosis on patients with NAFLD [6, 7], but it performs poorly in patients with high BMI due to the thick layer of subcutaneous adipose tissue [8]. The XL probe has been developed to overcome the issue of liver stiffness and CAP measurement in overweight patients. The XL probe takes measurements at greater depth then the M probe (i.e. 35–75 vs. 25–65 mm) using a lower-frequency ultrasound transducer (2.5 vs. 3.5 MHz) to increase ultrasound penetration. However, the XL probe was not initially developed to address morbidly obese patients who generally have a probe-to-capsula distance (PCD) greater than 35 mm. A thick layer of subcutaneous adipose tissue was associated with overestimation of liver steatosis by CAP measurement using M probe [8]. Measuring deeper into the liver may theoretically avoid this overestimation. In this context, the aim of our study was to assess whether CAP is overestimated using the standard XL probe in patients with morbid obesity, and in the case of an overestimation, to reprocess the data at a greater depth to obtain the appropriate CAP (CAPa).

## Patients and methods

### Study population

We conducted an observational prospective cohort study including consecutive patients admitted to our institution to undergo sleeve gastrectomy between July 2015 and July 2017. Patients were eligible for inclusion if they: 1) were severely obese [body mass index (BMI) ≥ 35 kg/m^2^] with comorbid conditions or morbidly obese alone (BMI ≥ 40 kg/m^2^) and were not responding to medical treatment; 2) had no medical or psychological contraindications for bariatric surgery; 3) had no current excessive drinking as defined by an average daily consumption of more than 20g alcohol/day for women and more than 30g alcohol/day for men; 4) had negative screening results for chronic liver disease nonrelated to obesity; 5) had a liver biopsy performed during the surgery; and 6) had a CAP measurement performed with transient elastography simultaneously with LSM measurement during the 15 days preceding liver biopsy. Liver biopsy was recommended in patients with ultrasound results suggestive of liver steatosis or liver dysmorphia and/or abnormal liver tests and/or a macroscopically abnormal liver as observed by the surgeons. All of these patients underwent a laparoscopic single port sleeve gastrectomy. Written informed consent was obtained from all participants. The study did not included minors and was conducted in accordance with the French law concerning medical investigations (Huriet Law) and the Helsinki declaration. The study protocol and the consent procedure were approved by the ethics committee of the Bicêtre Hospital.

### CAPa measurement

CAP was assessed simultaneously with liver stiffness using FibroScan (EchoSens, Paris, France) by trained operators in accordance with the manufacturer’s instructions. Ten successful acquisitions were performed each time. Patients were likely to have a probe-to-capsule distance of more than 35 mm and we therefore used the XL probe for the entire cohort. CAP values are expressed as the median and interquartile range (IQR) [in decibels per meter (dB/m)] of all valid measurements obtained. The characteristics of XL probe were as follows: central ultrasound frequency 2.5 MHz, ultrasound transducer focal length 50 mm, external diameter of the tip of the probe 12 mm, vibration amplitude (peak to peak) 3mm, and measurement depths 35–75mm. The operators were blind to all patient data.

An overestimation of CAP in obese patients may be because of the CAP recordings not having been made at a sufficient depth. CAP value is likely to be overestimated when using the XL probe if the PCD is greater than 35 mm. Therefore, all patient files were reprocessed retrospectively by a new algorithm, blinded to the patients’ clinical data. The algorithm automatically assessed the PCD by analyzing the echogenicity of ultrasound signals on the time-motion mode. The calculated depth was deduced of this probe-to-capsule distance and in the case of a distance superior to 35 mm, the algorithm automatically selected a deeper measurement (either 40–80 or 45–85 mm) for estimation of an appropriate CAP (CAPa) value. When PCD was less than 35 mm, the measured CAP was considered as appropriated (CAPa) and no further reprocessing was performed.

### Liver biopsy

Liver biopsies were performed during the laparoscopic single port sleeve gastrectomy as previously described [6]. A single experienced pathologist evaluated liver biopsies blinded to clinical, biological and CAP data. As previously described [9], liver steatosis was evaluated as a percentage and scored as 0 (absent) if below 5%, as 1 (mild) if 5–33%, as 2 (moderate) if 34–66%, and as 3 (severe) if more than 66%. Hepatocyte ballooning was classified as 0 (none), 1 (a few balloon cells), or 2 (prominent balloons). Foci of lobular inflammation were graded as 0 (no foci), 1 (<2 foci), 2 (2–4 foci), and 3 (>4 foci). As described by Kleiner et al [9], NAFLD activity score (NAS) was defined as the sum of the scores for steatosis (0–3), lobular inflammation (0–3), and ballooning (0–2); the total score thus ranged from 0 to 8 [9]. NASH was defined as a NAS of at least 5. Brunt–Kleiner classification was used to evaluate fibrosis: stage 0 (none), 1 (perisinusoidal or portal fibrosis), 2 (perisinusoidal and portal fibrosis without bridging), 3 (bridging fibrosis), or 4 (cirrhosis) [9]. This study was performed in accordance with the Helsinki declaration. The protocol was approved by the ethics committee of Bicêtre Hospital. All patients gave written informed consent.

### Statistical analysis

Quantitative variables are expressed as means ± standard error of the mean (SEM). χ^2^-Tests were used for comparisons of qualitative variables. Student’s t-test was used for comparisons of normally distributed quantitative variables, and the Mann–Whitney test or the Kruskal–Wallis test was used to compare quantitative variables that were not normally distributed. The Bonferroni multiple-comparison procedure was used. We evaluated simple correlations between variables with Spearman’s rank correlation tests. We then identified independent relationships between variables with a P value less than 0.05 in univariate analysis and CAP, through multiple linear regression analysis. The diagnostic performance of CAP was determined for each histological grade of steatosis by calculating the area under the receiver operating characteristic curve (AUROC) with an empirical nonparametric method, as described by DeLong et al. [10]. Sensitivity, specificity, positive predictive value (PPV) and negative predictive value (NPV) were calculated. The accuracy of CAPa at optimal thresholds was defined by the maximum Youden index. For each optimal cut-off value, the sensitivity, specificity, PPV, NPV and likelihood ratio (LR) were calculated. The Obuchowski measure was assessed, taking the distribution of steatosis grade in the cohort into account. The Obuchowski measure is a multinominal version of the AUROC designed for situations characterized by a nonbinary gold standard [11]. The Obuchowski measure overcomes both the spectrum effect and the ordinal scale. It can be interpreted as the probability of CAPa correctly ranking two randomly chosen patient samples from different steatosis grades according to the weighting scheme, with a penalty for the misclassification of patients. For this analysis, weighting was based on the relative distribution of steatosis grades in the cohort. The Obuchowski measure was assessed with a penalty function similar to that described by Lambert et al. [12]. This penalty function is proportional to the difference in units between grades. Using four categories for the gold standard outcome (steatosis grade), the estimated AUROC of diagnostic tests for differentiating between categories, the Obuchowski measure is a weighted average of the N(N− 1)/2 = 6 different AUROCs corresponding to all the pairwise comparisons between two of the N categories. Each pairwise comparison is weighted to take into account the distance between steatosis grades (i.e. the number of units on the ordinal scale). The penalty function was 0.33 when the difference between steatosis grades was 1, 0.67 when the difference between grades was 2, and 1 when the difference between grades was 3. We used Number Cruncher Statistical Systems software, version 9.0.14 [13] and R software for Obuchowski measures [14].

## Results

### Characteristics of the patients

A total of 257 obese patients fulfilled the inclusion criteria. Eight patients were excluded from the study due to unsuitable liver biopsy or unreliable CAP measurement. Most of the 249 included patients were female (79%), the mean age was 41.2±0.77 years and the BMI was 43.8±0.4 kg/m^2^. Type 2 diabetes, hypertension and dyslipidemia were present in 21.7%, 29.7% and 29% of included patients respectively. Steatosis was present in 84% of patients and one fifth of the included patients had a fibrosis score ≥2. Using Kleiner’s classification, 43% of patients were found to have NASH (NAS≥5). Characteristics of included patients are reported in Table 1.

**Table 1:** Characteristics of the patients included in the study.

Characteristics	n = 249
**Age (years)**	41.2±0.77
**BMI (kg/m2)**	43.8 ±0.4
**Female**	197 (79.1%)
**Type 2 diabetes**	54 (21.7%)
**Hypertension**	74 (29.7%)
**Dyslipidemia**	72 (29%)
**ALT (IU/L)**	45±2.4
**AST (IU/L)**	31.3±1.4
**GGT (IU/L)**	46±2.7
**Fasting blood glucose (mmol/L)**	6.1±0.2
**HbA1c (%)**	5.9±0.1
**Blood insulin (μUI/ml)**	28.2±1.2
**Total cholesterol (mmol/L)**	5.3±0.07
**Triglycerides (g/L)**	1.5±0.07
**HDL-cholesterol (mmol/L)**	1.3±0.04
**Uric acid (μmol/L)**	355.8±5.4
**Ferritin (μg/L)**	141.6±9.9
**CRP (mg/L)**	12±0.6
**Albumin (g/L)**	40.7±0.2
**Prothrombin Index (%)**	97±0.3
**Platelet number (x10^3^/mm^3^)**	266±3.8
**CAP (dB/m)**	318.7±4
**CAPa (dB/m)**	293±3.5
**Steatosis**	
**<5% (S0)**	39 (15.7%)
**5–33% (S1)**	65 (26.1%)
**34–66% (S2)**	51 (20.5%)
**>66% (S3)**	94 (37.7%)
**Activity score (NAS)**	
**NASH (NAS≥5)**	107 (43%)
**Non-NASH**	142 (57%)
**Fibrosis**	
**No fibrosis (F0)**	39 (15.9)
**Perisinusoidal or periportal fibrosis (F1)**	155 (63.3%)
**Perisinusoidal and periportal fibrosis (F2)**	39 (15.9%)
**Septal or bridging fibrosis (F3)**	11 (4.5%)
**Cirrhosis (F4)**	1 (0.4%)

**Note**: Results are given as the mean ± standard error of the mean or %.

**Abbreviations**: BMI, body mass index; AST, aspartate aminotransferase; ALT, alanine aminotransferase; GGT, gamma glutamyl; HbA1c, glycated hemoglobin; HDL, high density lipoprotein; CRP, C reactive protein; CAP, controlled attenuation parameter; CAPa, appropriate controlled attenuation parameter; NAS, nonalcoholic fatty liver disease activity score; NASH, nonalcoholic steatohepatitis.

### Analysis of patient files for CAP adaptation

**Fig 1:**
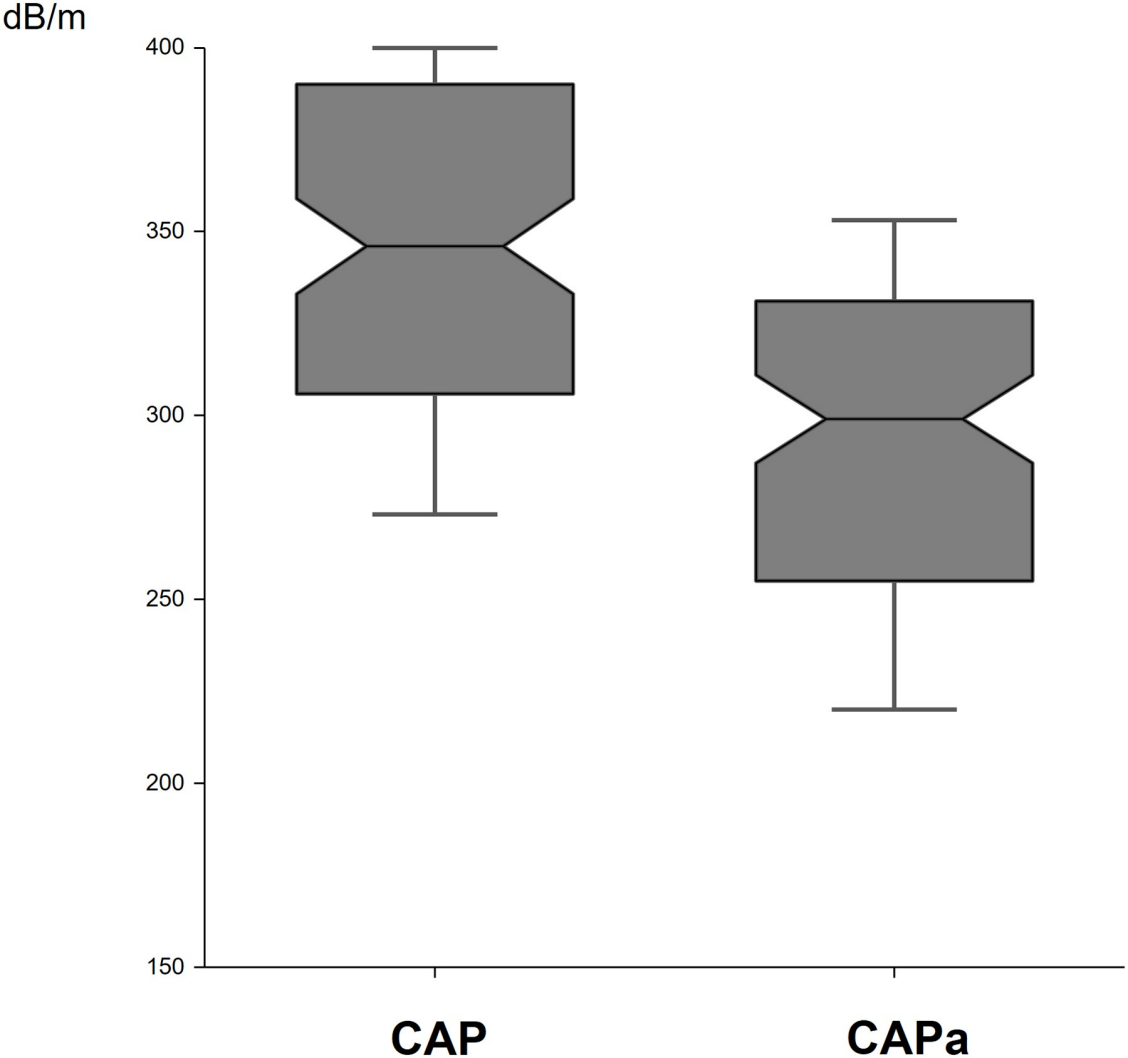
Controlled attenuation parameter before (CAP) and after adaptation (CAPa) of measurement depth in the group of patients with probe-to-capsula distance (PCD) >35 mm. Notched box plots showing CAP and CAPa. The line in the box indicates the median; the height of each box is the median ± (1.57 interquartile range/√n), used to assess the 95% confidence interval around group medians. Differences are considered to be significant if the shaded boxes do not overlap (p< 0.05). The horizontal lines above and below each box indicate the interquartile range (from the 25th to the 75th percentile), and the vertical lines at the ends of the box encompass the adjacent values (upper: 75th percentile + 1.5 times the interquartile range; lower: 25th percentile—1.5 times the interquartile range).

After analysis of all patient files, the CAP was considered to be appropriate for 119 patients for whom the PCD was less than 35 mm. For these patients, CAP was not recalculated. For the other 130 patients the CAP recording was not performed at a sufficient depth (PCD greater than 35 mm). Therefore, after reprocess, the appropriate CAP (CAPa) was obtained between 40 and 80 mm or between 45 and 85 mm. In these group of patients, the CAPa reprocessed at the adapted depth was significantly lower (298±3.9) than the CAP measured between 35 and 75 mm (340±4.2) (p< 0.0001) (Fig 1). Furthermore, the reprocessed CAP (CAPa) was not significantly different of the CAP measured in the group of patients with PCD<35 mm (298±3.9 versus 303.4±5.5; p = 0.16). Characteristics of patients with PCD <35 mm and >35 mm are reported in Table 2.

**Table 2:** Characteristics of the patients with probe-to capsula distance (PCD) <35 mm versus >35 mm.

Characteristics	PCD <35 mm (n = 119)	PCD >35 mm (n = 130)	p
**Age (years)**	42.5±1	39.4±1.3	NS
**BMI (kg/m2)**	42.4±0.5	45.7±0.6	<0.00001
**Female**	121 (82.9%)	76 (73.8%)	NS
**Type 2 diabetes**	32 (21.9%)	22 (21.4%)	NS
**Hypertension**	46 (31.5%)	18 (27.2%)	NS
**Dyslipidemia**	40 (27.6%)	32 (31.1%)	NS
**ALT (IU/L)**	30.6±1.3	32.2±2.8	NS
**AST (IU/L)**	43.1±2	47.7±5	NS
**GGT (IU/L)**	43.7±3	49.2±5	NS
**Fasting blood glucose (mmol/L)**	6±0.3	6.2±0.2	NS
**HbA1c (%)**	5.8±0.1	6±0.1	NS
**Blood insulin (μUI/ml)**	25.3±1.4	32.4±2	<0.01
**Triglycerides (g/L)**	1.5±0.1	1.6±0.1	NS
**Uric acid (μmol/L)**	339.6±6.6	378.7±8.6	<0.001
**Ferritin (μg/L)**	129.5±11.3	158.8±17.6	NS
**CRP (mg/L)**	10.8±0.7	13.7±1.1	NS
**CAP (dB/m)**	303.4±5.5	340.5±5.1	<0.00001
**Steatosis**			
**<5% (S0)**	26 (17.8%)	13 (12.6%)	NS
**5–33% (S1)**	34 (23.3%)	31 (30.1%)	NS
**34–66% (S2)**	33 (22.6%)	18 (17.5%)	NS
**>66% (S3)**	53 (36.3%)	41 (39.8%)	NS
**Activity score (NAS)**			
**NASH (NAS≥5)**	62 (43.8%)	43 (41.7%)	NS
**Non-NASH**	84 (56.2%)	60 (58.3%)	NS
**Fibrosis**			
**Septal or bridging fibrosis (F3) or cirrhosis (F4)**	4 (2.8%)	7 (7%)	NS

**Note**: Results are given as the mean ± standard error of the mean or %.

**Abbreviations**: BMI, body mass index; AST, aspartate aminotransferase; ALT, alanine aminotransferase; GGT, gamma glutamyl; HbA1c, glycated hemoglobin; HDL, high density lipoprotein; CRP, C reactive protein; CAP, controlled attenuation parameter; NAS, nonalcoholic fatty liver disease activity score; NASH, nonalcoholic steatohepatitis.

### Relationship of BMI and histological parameters to CAP values

In univariate analysis, CAP was positively correlated with the BMI (r = 0.18; p<0.01), steatosis stage (r = 0.55; p<0.0001), fibrosis score (r = 0.14; p = 0.03) and NAS score (r = 0.48; p<0.0001). Multiple linear regression analysis revealed that both BMI (regression coefficient = 2.05; p<0.001) and hepatic steatosis (regression coefficient = 15.05; p = 0.0001) were still independently associated with CAP values.

After reprocessing the CAP in patients with PCD > 35 mm, only steatosis stage (r = 0.58; p<0.0001) and NAS score (r = 0.52; p<0.0001) were positively correlated with CAP values in univariate analysis. In multiple linear regression analysis, steatosis stage (regression coefficient = 15.52; p< 0.0001) was the only parameter independently correlated with CAP values.

### The impact of CAP measurement at an appropriate depth on the diagnostic performance

For discriminating steatosis (S≥1), moderate to severe steatosis (S≥2) and severe steatosis (S = 3) the AUROCs of CAP were higher in the group of patients with PCD <35 mm (119 patients) than in those with PCD ≥35 mm (130 patients): 0.84 versus 0.80 (p = 0.62), 0.89 versus 0.69 (p<0.001) and 0.87 versus 0.67 (p = 0.0019), respectively.

### Diagnostic value of CAPa for assessing hepatic steatosis

**Fig 2:**
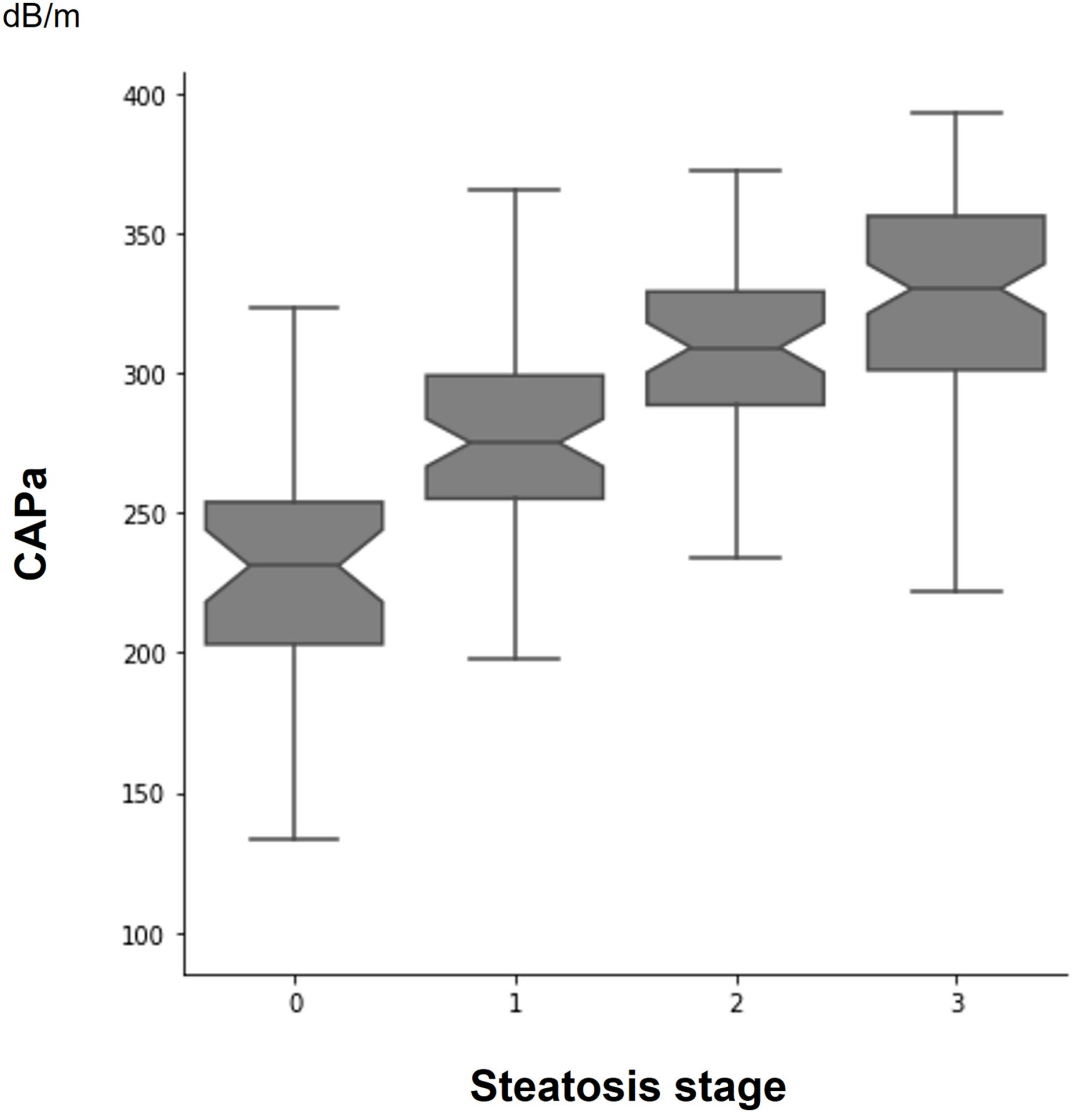
Distribution of appropriate controlled attenuation parameter (CAPa) according to steatosis stage. Notched box plots showing the relationship between steatosis stage and CAPa. The line in the box indicates the median; the height of each box is the median ± (1.57 interquartile range/√n), used to assess the 95% confidence interval around group medians. Differences are considered to be significant if the shaded boxes do not overlap (p< 0.05). The horizontal lines above and below each box indicate the interquartile range (from the 25th to the 75th percentile), and the vertical lines at the ends of the box encompass the adjacent values (upper: 75th percentile + 1.5 times the interquartile range; lower: 25th percentile—1.5 times the interquartile range).

The CAPa includes the unprocessed CAP (measured CAP) for patients with probe-to-capsula distance <35 mm and reprocessed CAP for patients with probe-to-capsula distance >35 mm. CAPa values were between 100 and 393 dB/m with a median of 298 dB/m. The mean value of CAPa measurements progressively increased with the stage of hepatic steatosis: S0–232±8 dB/m, S1–274±6 dB/m, S2–306±5 dB/m, S3–324±5 dB/m. Furthermore, the CAPa values were significantly different between each steatosis stage (Fig 2). CAPa was also highly correlated with steatosis stage (r = 0.58; p<0.0001) (Fig 2).

**Fig 3:**
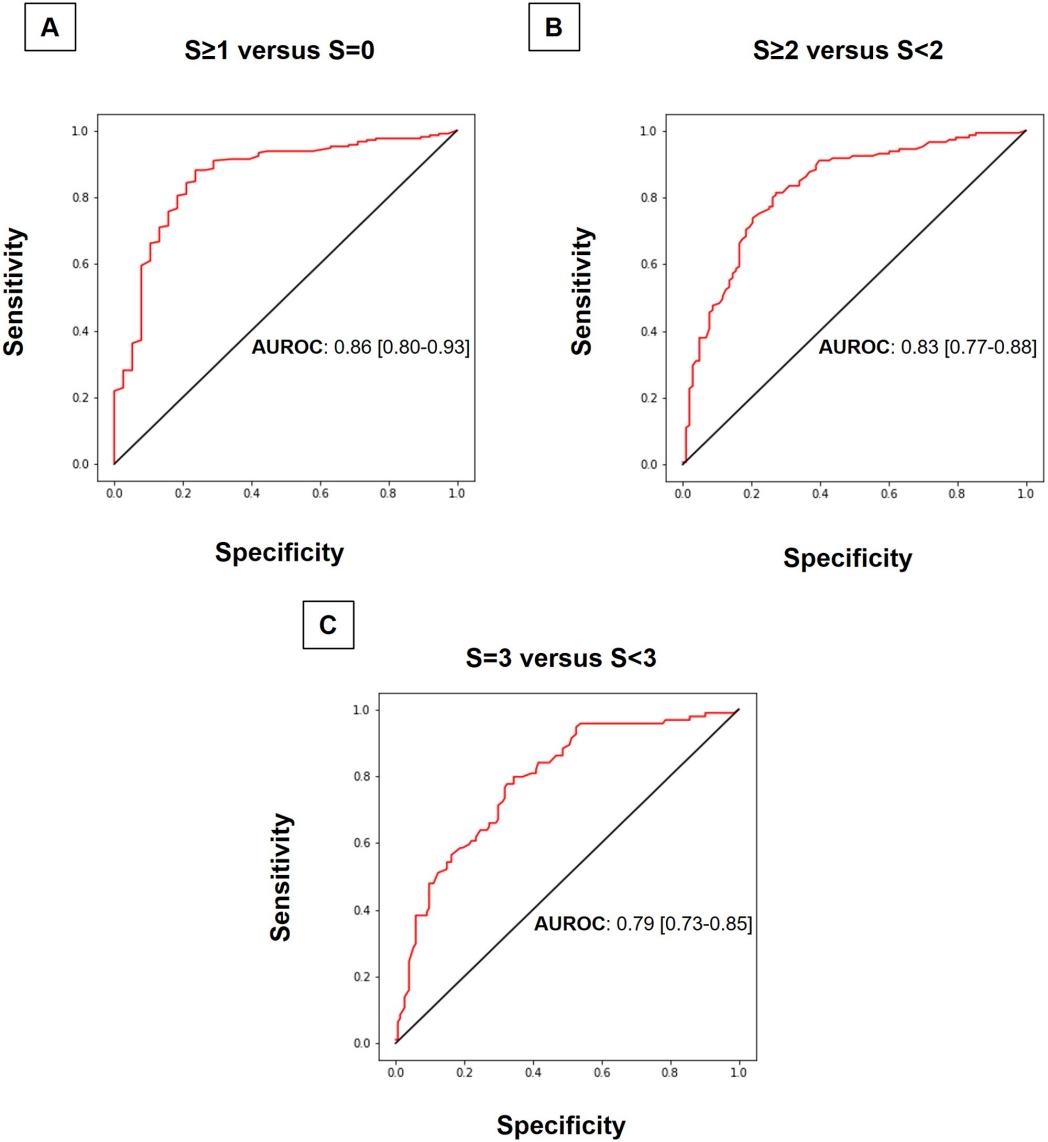
ROC curves of appropriate controlled attenuated parameter (CAPa) for the detection of (A) steatosis (S≥1), moderate to severe steatosis (S≥2) and severe steatosis (S = 3). The diagonal line represents detection achieved by chance alone (AUROC = 0.50); the ideal AUROC is 1.00.

For discriminating patients with steatosis (S≥1) of those without steatosis (S = 0), the AUROC of CAPa was 0.86 (95% confidence interval [CI] 0.80–0.93) (Fig 3A). For discriminating between moderate or severe steatosis and mild or no steatosis, the AUROC for CAPa was 0.83 (95% CI 0.77–0.88) (Fig 3B). The diagnosis accuracy of CAPa for differentiating S3 (severe steatosis) from S0-S1-S2 was 0.79 (95% CI 0.73–0.85) (Fig 3C). For the diagnosis of steatosis, the Obuchowski measure, with a penalty function as described by Lambert *et al*. [12], was 0.90±0.013. Furthermore, diagnosis value of CAPa was better than that of unprocessed CAP for differentiating S0-S1 from S2-S3 [0.83 (95% CI 0.77–0.88) versus 0.79 (95% CI 0.73–0.85); p = 0.07], but not for differentiating S0 from S1-S2-S3 [0.85 (95% CI 0.77–0.91) versus 0.85 (95% CI 0.77–0.91)] or S3 from S0-S1-S2 [0.78 (95% CI 0.71–0.83) versus 0.77 (95% CI 0.70–0.82)].

The optimal cut-off value for CAPa was 255 dB/m for steatosis (S≥1 versus S = 0), 288 dB/m for moderate to severe steatosis (S≥2 versus S<2), and 297 dB/m for severe steatosis (S = 3 versus S<3) (Table 3). For the diagnosis of steatosis, a cut-off of 255 dB/m gave an excellent PPV of 95% (Table 3).

**Table 3:** Diagnostic performance of appropriate controlled attenuation parameter (CAPa) for the detection of steatosis stage.

Steatosis	Optimal cut-off (dB/m)	Sensitivity	Specificity	PPV	NPV	LR
S0 vs. S1-3	255	0.88	0.76	0.95	0.54	3.7
S0-1 vs. S2-3	288	0.81	0.73	0.81	0.74	3
S3 vs. S0-2	297	0.80	0.66	0.59	0.84	2.3

**Abbreviations**: PPV, positive predictive value; NPV, negative predictive value; LR, likelihood ratio.

## Discussion

The PCD was greater than 35 mm in more than a half of our cohort of patients with morbid obesity. In these patients, the CAP measured at an adjusted depth was lower than that measured at the standard depth of XL probe. Our results suggest that CAP measurements are probably overestimated in morbidly obese patients despite the use of the XL probe. The poor performance and high failure rate of CAP measurement in patients with high BMI was already proved by studies using M probe [8, 15]. Furthermore, CAP measured by M probe showed a low diagnostic value in patients with PCD greater than 25 mm [16]. In accordance with these data, our study finds a poor CAP performance in differentiating steatosis using XL probe in patients with PCD greater than 35 mm. The PCD can be very large in severely obese patients because of the thick layer of subcutaneous adipose tissue. The subcutaneous fat is involved in the measurement using the XL probe in patients with a PCD greater than 35 mm, strengthening the degree of attenuation and overestimating CAP. In a previous study, we showed that presence of nonhepatic tissue in the volume explored by the XL probe also attenuates the transmission of shear waves into the liver and the ultrasonic signals used to measure their speed of propagation, leading to an overestimation of stiffness values [17].

In our study, both BMI and steatosis were independently associated with CAP measured by XL probe. After adjustment of measurement depth, steatosis was the only parameter independently correlated with CAPa. The thick layer of subcutaneous adiposity can account at least partially for the association between CAP and BMI. A previous study using the M probe also showed an independent association between BMI and CAP which still persisted in patients with steatosis less than 5% [18]. Overall, these data suggest the contribution of subcutaneous adipose tissue to CAP overestimation in patients with morbid obesity.

We also show that CAPa has very good diagnostic performance for steatosis in patients with morbid obesity candidates to bariatric surgery. The AUROC of CAPa for the diagnosis of steatosis (S≥1) and moderate to severe steatosis (S≥2) was 0.86 and 0.83, respectively. Our results are in accordance with the recent individual patient data meta-analysis evaluating CAP performance using M probe which showed AUROC values of 0.82 and 0.86 for steatosis and moderate to severe steatosis, respectively [19]. Patients included in this large meta-analysis had chronic liver disease of various etiologies (20% of NAFLD patients) and a much lower BMI (25±3.9 kg/m^2^) comparing to our cohort (44±6.2 kg/m^2^). Furthermore, a study including 236 patients with chronic liver disease found that CAP obtained using the XL probe have similar performance as the M probe for the estimation of hepatic steatosis, with AUROC values >0.80 [20]. Disease prevalence in the studied cohort influences diagnostic test probabilities [21]. In our cohort, the prevalence of steatosis and moderate to severe steatosis was 84% and 58%, respectively. By using Obuchowsky measure to evaluate diagnosis accuracy we also took into account the distribution of steatosis stages (diagnosis accuracy: 0.90). Overall, the present study shows that CAP has a very good performance for the diagnosis of steatosis even in severely or morbidly obese patients, although the correct measurement of CAP in these patients only requires adaptation of the measurement depth farther below the skin surface.

The optimal cut-offs for CAPa in our study were 255 dB/m, 288 dB/m and 297 dB/m for steatosis (S≥1), moderate to severe steatosis (S≥2) and severe steatosis (S = 3), respectively. The cut-off for steatosis was close to that reported by the recent individual patient data meta-analysis on CAP performance using M probe (248 dB/m) [19]. Nevertheless, our cut-off for moderate to severe steatosis and severe steatosis was slightly higher than that reported in the meta-analysis (268 dB/m and 280 dB/m, respectively). In our previous study including 317 patients with morbid obesity (retrospective cohort: 194 patients; prospective cohort: 123 patients), the cut-offs of CAP using XL probe were superior to 300 dB/m for all steatosis grades [6]. In a study of 261 patients with NAFLD, de Lédinghen and colleagues reported cut-off values for the detection of moderate to severe steatosis and severe steatosis, at 310 and 311 dB/m, respectively [22]. CAP cutoffs may differ between studies, probably as a function of liver disease etiology, the prevalence of different grades of steatosis in the study group, and the degree of subcutaneous adiposity. The results of our study suggest that CAP cut-offs using XL probe are close to those reported for M probe, but requires adaptation of measurement depth in patients with severe and morbid obesity. Nevertheless, the best cutoff values of CAP using XL probe remain to be defined.

For defining NAFLD, there must be evidence of hepatic steatosis and lack of secondary causes of hepatic fat accumulation. Accurate diagnosis of steatosis in patients with metabolic risk factors is therefore essential of early diagnosis of NAFLD. In our cohort of patients with morbid obesity, a cut-off for CAPa of 255 dB/m for the diagnosis of steatosis (S≥1) gave an excellent PPV of 95%, but a NPV of only 54%. Good PPVs >90% and low NPV were obtained in the other published studies evaluating NAFLD patients [4, 7]. These data suggest that CAP is a reliable test for the diagnosis of steatosis in NAFLD patients. CAP may also be a promising tool to follow-up NAFLD patients. However, the utility of noninvasively quantifying of steatosis by CAP in patients with NAFLD in clinical studies or routine clinical care remains to be determined.

Our study may have several limitations. First, for technical reasons, liver biopsy was performed on the left lobe during bariatric surgery, whereas CAP was performed on the right lobe of the liver. Nevertheless, a previous study of morbidly obese patients showed uniform histological findings across the hepatic lobes using paired liver biopsies [23]. Second, the time between liver biopsy and CAP measurement was up to 15 days. It is well known that steatosis may change quickly with lifestyle or drug interventions. However, the patients included in our study failed all lifestyle interventions prior to surgery and did not lost weight. Furthermore, there was no lifestyle intervention during the 15 days before surgery.

. In conclusion, the results of our study show that CAP can be correctly performed in morbidly obese patients and is a reliable diagnostic test for steatosis. However, an overestimation of the CAP using the XL probe is observed in 50% of patients with morbid obesity. The correct measurement of CAP in these patients only requires adaptation of measurement depth farther below the skin surface. The possibility to select the appropriate measurement depths by the operator in patients with skin-to-capsula distance greater than 35 mm could overcome this limitation in the future.
